# Work, Wealth Death

**Published:** 1870-04

**Authors:** 


### WORK, WEALTH DEATH.

To work hard, get rich, and die prematurely, is the sad history of millions. To work hard, accumulate money, and live healthily and long, is the glad record of the few.

One man is at his business early; drives incessantly until the middle of the afternoon; won't take time to eat at midday; or if he does, it is to take his lunch at a swallow, wash it down with a glass of spirits, to go ahead with greater desperation to make up the loss of time. At five or six o'clock he sits down to his dinner in a state of exhaustion, body worn out, spirits depressed, and almost famishing for food, which he hastily crams into his stomach until he is very much in the condition of a gorged anaconda; in due time, he retires to bed; not to sweet and heavenly sleep, but to tumultuous tossings, disturbing dreams, and an uneasy, weary waking. But he makes money and dies before fifty.

Another is at his place of business while the former is still in bed, leaves it at dark, grows rich, and lives healthfully until fourscore. The difference is in two things; the latter takes a leisure and full dinner at noon; eats nothing more that day; is always in bed by ten o'clock, sleeps soundly until six in the morning, and is wide awake to business the whole of the day following. I know a man who has fallen into this manner of life from principles of living learned from *Hall's Journal of Health*, in the course of its publication. A very few years ago he thought he was doing well to sell a hundred dollars worth of goods in a day. Last Saturday he sold over eight thousand dollars worth at retail. In over twelve hundred thousand dollars worth of sales, he has not lost a hundred and fifty, all told, from all causes, whether from miscarriages of parcels or otherwise.

Go into his place of business, he is always on hand; nothing is done by the hundreds of his men, without the cognizance of his watchful eye; always prompt, always courteous, always accommodating, his mind always on the alert, he is master of the situation, master of his business, and is coining money. The secret—abundant sleep, living wisely; in fact he is the model, drawn from our book, on "HEALTH BY GOOD LIVING," a living demonstration of its literal truthfulness. Would the reader like to make his acquaintance, and thus verify these statements, go and see Baldwin the clothier, N. E. corner of Broadway and Canal Street, New York. Then come back and buy our book, one for yourself and one for each of your children.

